# Diverse modes of chromosome terminal deletion in spontaneous canavanine-resistant *Schizosaccharomyces pombe* mutants

**DOI:** 10.17912/micropub.biology.001132

**Published:** 2024-02-05

**Authors:** Xiao-Hui Lyu, Fang Suo, Wen Li, Guo-Song Jia, Yu-Sheng Yang, Li-Lin Du

**Affiliations:** 1 National Institute of Biological Sciences, Beijing, China; 2 Tsinghua Institute of Multidisciplinary Biomedical Research, Tsinghua University, Beijing, China

## Abstract

Canavanine resistance has been used to analyze mutation rates in the fission yeast *Schizosaccharomyces pombe* . However, the genetic basis of canavanine resistance in this organism remains incompletely understood. Here, we performed whole genome sequencing on five spontaneously arising canavanine-resistant *S. pombe* mutants, including the *can2-1* mutant isolated in the 1970s. This analysis revealed that three mutants, including *can2-1* , experienced terminal deletions of the left arm of chromosome II, leading to the loss of multiple amino acid transporter genes. Interestingly, these three mutants underwent chromosome terminal deletion through distinct mechanisms, including homology-driven translocation, homology-independent chromosome fusion, and de novo telomere addition. Our findings shed new light on the genetic basis of canavanine resistance and mechanisms underlying chromosome terminal deletions in fission yeast.

**Figure 1. Chromosome II left arm terminal deletions in canavanine-resistant S. pombe mutants:**
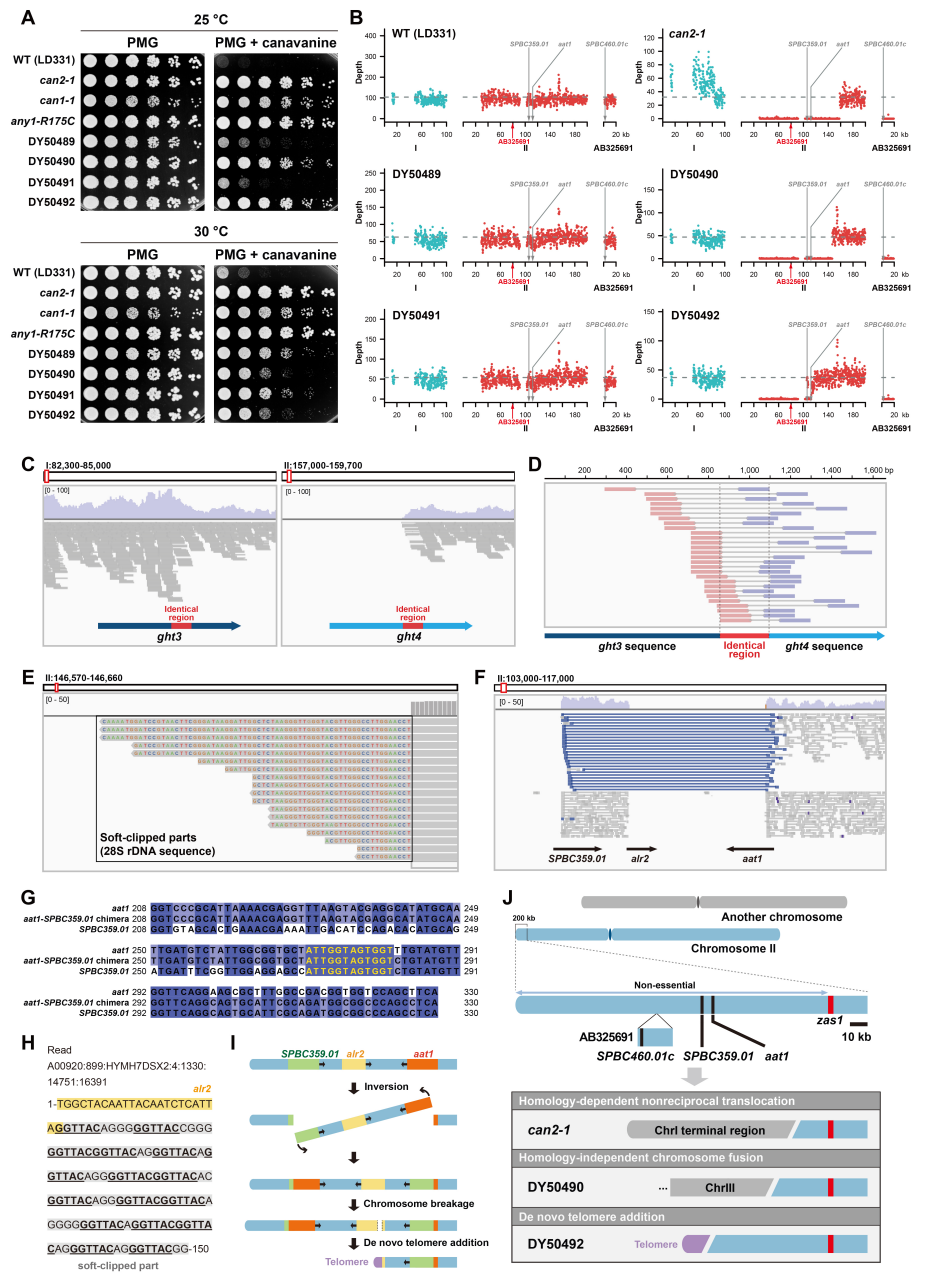
**(A)** Spot assay showing the canavanine resistance of a *can1-1* strain (DY7144), a *can2-1* strain (DY49525), an *any1-R175C* strain (DY50493) derived from LD331, and four spontaneous canavanine-resistant strains (DY50489–DY50492) derived from LD331. The concentration of canavanine in canavanine-containing plates is 5 μg/mL. **(B)** Sliding-window read depth plots showing the chromosome II left arm terminal deletions in the *can2-1* strain, DY50490, and DY50492 and the chromosome I left arm terminal duplication in the *can2-1* strain. The first 100 kb of chromosome I, the first 200 kb of chromosome II, and the whole AB325691 contig are shown in the plots. The horizontal dashed line in each plot represents the average depth across the genome. AB325691 is a gap-filling contig (Sasaki et al., 2008) and the corresponding gap in chromosome II is indicated by a red arrow. The positions of three amino acid transporter genes are indicated by grey arrows. Sliding window bin size = 200 bp. Only bins in unique regions of the genome are analyzed. **(C)** Genome browser views showing the reads of the *can2-1* strain mapped to the *ght3* locus and the *ght4* locus. The CDSs of *ght3* and *ght4* are shown as arrows. **(D)** Read pairs supporting the presence of a *ght3-ght4* chimera in the *can2-1* strain. The forward reads of the two read pairs at the bottom have one nucleotide outside of the identical region. **(E)** Soft-clipped reads mapped to the deletion junction in DY50490 contain the sequence of the 28S rDNA within their soft-clipped parts. **(F)** Genome browser view showing the reads of DY50492 mapped to the region surrounding the deletion junction. Mapped paired-end reads are shown as connected pairs. Discordant read pairs (colored blue) reveal an inversion between *SPBC359.01* and *aat1* . The CDSs of *SPBC359.01* , *alr2* , and *aat1* are shown as arrows. **(G)** Multiple sequence alignment of *aat1* (top), *SPBC359.01* (bottom), and the *aat1* - *SPBC359.01* chimera in DY50492 (middle). The 12-bp breakpoint is highlighted in yellow. **(H)** A soft-clipped read mapped to the deletion junction in DY50492. In its soft-clipped part (colored grey), there are multiple copies of the core telomeric repeat sequence GGTTAC (underlined). **(I)** Diagram showing that the sequence loss in DY50492 is due to a complex rearrangement combining an inversion and a chromosome terminal deletion healed by de novo telomere addition. The diagram is not drawn to scale. **(J)** Diagram summarizing the mechanisms underlying the chromosome terminal deletions in the canavanine-resistant strains analyzed in this study. Only chromosome II and its enlarged terminal region are drawn to scale.

## Description

Canavanine, an arginine analog, can enter cells through amino acid transporters (Grenson et al., 1966; Fantes and Creanor, 1984) . Once inside cells, canavanine becomes incorporated into proteins, impairing their function and causing cytotoxicity (Rosenthal, 1991) . In the budding yeast *Saccharomyces cerevisiae* , spontaneously arising canavanine-resistant mutations are exclusively those disabling the arginine transporter gene *CAN1* (Whelan et al., 1979) . Since *CAN1* is located in a terminal, nonessential region of chromosome V, canavanine resistance can result from terminal deletion of this chromosome­—a type of gross chromosomal rearrangement (GCR) (Chen et al., 1998; Chen and Kolodner, 1999) . The well-defined genetic basis of canavanine resistance in *S. cerevisiae* has led to the widespread use of canavanine resistance as an assay to analyze the rates and spectra of both small mutations and GCRs in this organism (Lang and Murray, 2008; Putnam and Kolodner, 2017; Jiang et al., 2021) .

Canavanine resistance has also been used to analyze mutation rates in the fission yeast *Schizosaccharomyces* *pombe* (Kaur et al., 1999; Tanaka et al., 2004; Sabatinos et al., 2013) . However, unlike in *S. cerevisiae* , the genetic basis of canavanine resistance in *S. pombe* is not fully understood. The first reported canavanine-resistant mutants in *S. pombe* are two spontaneous mutants *can1-1* and *can2-1* isolated in the 1970s (Kohli et al., 1977; Fantes and Creanor, 1984; Jürg Kohli, personal communication). Recently, the *can1-1* mutation was identified as a gain-of-function mutation (R175C) in the *any1* gene, which encodes a ubiquitin ligase that negatively regulates amino acid transporters (Yang et al., 2022; Saada et al., 2022; Pai et al., 2023) . Several other *S. pombe* genes, including *tsc1* , *tsc2* , *pas1* , and *cat1* , have been reported to cause canavanine resistance when individually deleted (van Slegtenhorst et al., 2004, 2005; Aspuria and Tamanoi, 2008) . However, it is unclear to what extent mutations in these genes are responsible for spontaneous canavanine resistance in *S. pombe* . The identity of the *can2-1* mutation has remained unknown.

During our investigation of the *can1-1* mutation (Yang et al., 2022) , we isolated four spontaneous canavanine-resistant mutants. They were derived from a wild-type *S. pombe* strain LD331. Among these four mutants, DY50489 and DY50491 showed stronger canavanine resistance at 30 °C than at 25 °C, while DY50490 and DY50492 exhibited stronger resistance at 25 °C than at 30 °C ( Figure 1A ). As a control, an *any1-R175C* knock-in mutant constructed in the LD331 background displayed greater canavanine resistance at both temperatures compared to the four mutants. A *can1-1* strain and a *can2-1* strain deposited by Jürg Kohli at the Yeast Genetic Resource Center of Japan (YGRC/NBRP) also showed canavanine resistance as expected.

We performed next-generation sequencing on the *can2-1* strain and the four LD331-derived canavanine-resistant mutants. No mutations were found in *any1* , *tsc1* , *tsc2* , *pas1* , and *cat1* . Remarkably, the *can2-1* strain, DY50490, and DY50492 each harbored a >100-kb terminal deletion on the left arm of chromosome II ( Figure 1B ). Even though the deletion junctions fall at different positions in these three strains, the deletions all result in the loss of more than one amino acid transporter gene. In the case of the *can2-1* strain and DY50490, three amino acid transporter genes *SPBC460.01c* , *SPBC359.01* , *aat1* are lost ( Figure 1B ). In the case of DY50492, due to a recombination between *SPBC359.01* and *aat1* , no intact *SPBC460.01c* , *SPBC359.01* , or *aat1* remains and an *aat1* - *SPBC359.01* chimera is present near a newly formed telomere and thus may be silenced (see more details below). The deletion of *aat1* is known to cause a reduction of arginine uptake by more than 50% (Nakase et al., 2012) . *SPBC460.01c* and *SPBC359.01* encode amino acid transporters closely related to Aat1 (Yang et al., 2022) . It is likely that the simultaneous loss of function of these amino acid transporter genes is the underlying cause of the canavanine resistance phenotype of these three strains. This conclusion is consistent with previous assignment of the *can2* locus to chromosome II by genetic mapping (Kohli et al., 1977) . The only mutation found in DY50489 was a missense mutation (A535T) in *rpc1* that encodes a subunit of RNA polymerase III, while the only mutation found in DY50491 was a missense mutation (P308S) in *drs1* that encodes aspartyl-tRNA synthetase. It remains to be determined whether these two mutations are related to the canavanine resistance phenotype.

We next investigated the mechanisms of chromosome II terminal deletions in the *can2-1* strain, DY50490, and DY50492 by inspecting the next-generation sequencing reads. The deletion junction in the *can2-1* strain is located in the middle of the *ght4* gene on chromosome II ( Figure 1C ). Interestingly, read depth analysis revealed a duplication of the left arm terminal sequence of chromosome I in this strain ( Figure 1B ). The duplication ends in the middle of the *ght3* gene ( Figure 1C ). *ght3* and *ght4* encode closely related hexose transporters (84% overall amino acid identity) (Heiland et al., 2000) . Moreover, a 240-bp sequence in these two genes share 100% nucleotide identity (codons 286–365, denoted as the “identical region” in Figures 1C and 1D). Because these two genes are both oriented in a telomere-to-centromere direction ( Figure 1C ), a nonreciprocal translocation occurring through homologous recombination between their identical regions could explain the terminal deletion on chromosome II and the terminal duplication on chromosome I. In support of this scenario, we identified read pairs that map to a *ght3* - *ght4* chimera, but not to *ght3* or *ght4* ( Figure 1D ). Collectively, these findings indicate that the chromosome II terminal deletion in the *can2-1* strain results from a homology-driven unbalanced translocation.

The deletion junction of DY50490 is located in the intergenic region between *SPBC1683.04* and *thi7* . Soft-clipped reads at the deletion junction indicate that the first undeleted nucleotide is at coordinate 146651 of chromosome II ( Figure 1E ). Strikingly, the soft-clipped parts of these reads match the sequence of the 28S rDNA gene, which normally resides in the two rDNA arrays located at the two ends of chromosome III ( Figure 1E ). The rDNA nucleotide immediately next to the first undeleted nucleotide of chromosome II is at position 2028 of the annotated 28S rDNA gene (GenBank accession Z19578.1). In the soft-clipped reads, the rDNA sequence is oriented with its telomere-proximal side facing the deletion junction, suggesting that chromosome II and chromosome III have fused at this junction. No microhomology was observed between the chromosome II sequence and the rDNA sequence flanking the fusion breakpoint. Thus, the chromosome II terminal deletion in DY50490 is likely due to a homology-independent chromosome fusion event. Because the dicentric chromosome resulting from such a fusion event is incompatible with cell viability, the fusion event presumably has been followed by either breakage of the fused chromosome or epigenetic inactivation of one of the two centromeres in the fused chromosome (Sato et al., 2012; Gu et al., 2022) .

Based on the read mapping result, DY50492 has undergone sequence loss in two regions: a chromosome II left arm terminal region telomeric to the amino acid transporter gene *SPBC359.01* and an approximately 5-kb region centromeric to *SPBC359.01* ( Figure 1F ). Discordant read pairs revealed that this dual loss pattern probably results from an inversion event followed by a chromosome terminal deletion event ( Figure 1F ). The two inversion junctions fall within *SPBC359.01* and *aat1* , which are homologous amino acid transporter genes with opposite orientations. Alignment of the coding sequences of *aat1* , *SPBC359.01* and the *aat1* - *SPBC359.01* chimera in DY50492 narrowed the inversion breakpoint to a 12-bp sequence identical between *SPBC359.01* and *aat1* ( Figure 1G ). The chromosome terminal deletion junction in DY50492 is located in the middle of *alr2* , the gene situated between *SPBC359.01* and *aat1* . The first undeleted nucleotide is at the 62nd position of the coding sequence of *alr2* . In the soft-clipped reads mapped to the deletion junction, the soft-clipped parts contain tandem copies of the core telomeric repeat sequence GGTTAC ( Figure 1H ) (Ares and Chakrabarti, 2008) , indicating that the deletion has been healed by de novo telomere addition. A schematic depicting the inversion and deletion events is shown in Figure 1I . We suspect that the *aat1* - *SPBC359.01* chimera in DY50492 may not be functional, because its close proximity to the telomeric repeats may result in its silencing.

Our findings indicate that the loss of function of three amino acid transporter genes ( *SPBC460.01c* , *SPBC359.01* , and *aat1* ) through chromosome II left arm terminal deletion may account for a significant fraction of spontaneous canavanine-resistant mutants in *S. pombe* . It is remarkable that the three chromosome terminal deletion mutants that we analyzed ( *can2-1* , DY50490, and DY50492) involve three distinct deletion mechanisms ( Figure 1J ). On the left arm of chromosome II, the most telomeric essential gene is *zas1* (Sasaki et al., 2013) , which is located about 71 kb centromeric to *SPBC359.01* ( Figure 1J ). Assuming that canavanine resistance requires the deletion junction to be on the centromeric side of *SPBC359.01* so that *SPBC460.01c* and *SPBC359.01* are lost and *aat1* is either lost or inactivated by telomeric silencing, but not too close (< 5 kb) to *zas1* to avoid its silencing, the deletion junction can fall anywhere within an approximately 66 kb region. The large size of this region may explain the abundance of chromosome II left arm terminal deletion mutants among spontaneous canavanine-resistant mutants and the diverse mechanisms underlying the deletions. Further study of spontaneous canavanine-resistant *S. pombe* mutants may provide additional insights into the mechanisms of chromosomal rearrangement.

## Methods

**Isolation of spontaneously arising canavanine-resistant mutants:** In our previous study (Yang et al., 2022) , we conducted *any1-R175C* knock-in by transforming a wild-type strain (LD331) with an *any1-R175C* PCR product and selecting canavanine-resistant clones. A no-DNA control transformation also yielded a small number of canavanine-resistant clones on PMG plates supplemented with 5 μg/mL canavanine. Four canavanine-resistant clones from this no-DNA control transformation were saved as DY50489–DY50492.

**Spot assay:** Cells were cultured to log phase in the YES medium and harvested by centrifugation. Cell pellets were washed three times with sterile water and resuspended to an OD _600_ of 0.4. 200 μL of each cell suspension was added to a 96-well plate and serial five-fold dilutions of each strain were prepared. Cell suspensions were spotted onto PMG plates with or without 5 μg/ml of canavanine using a pin tool and cultured at 30 °C for 108 hours. Images of plates were obtained using an Epson Perfection V800 Photo scanner.

**Whole-genome sequencing:** Illumina sequencing libraries were constructed using home-made Tn5 transposase as described previously (Tao et al., 2019) . Paired-end Illumina sequencing (2 x 150 bp) was performed on the Illumina NovaSeq 6000 system at Novogene (Beijing, China). The sequencing data have been deposited in the NCBI Short Read Archive (SRA) under BioProject accession PRJNA1062442. The sequencing data of LD331 was deposited under the strain name DY49197.

**Read mapping:** Illumina sequencing reads were cleaned using fastp (version 0.20.0) (Chen et al., 2018) and then mapped onto the reference genome of *S. pombe* using BWA-MEM (v0.7.17-r1188) (Li and Durbin, 2009) . Mapped reads were visualized using the Integrative Genomics Viewer (IGV) (v2.16.1) (Robinson et al., 2011) .

**Read-depth analysis** : Mappability was calculated by running GenMap version 1.3.0 using 100 bp k-mers with up to 2 mismatches (Pockrandt et al., 2020) . Genomic regions larger than 1 kb, where every position has a mappability value of one, were considered unique regions. For these regions, we plotted the average read depth in 200-bp sliding window calculated using samtools depth (version1.14) (Li et al., 2009) .

## Reagents

**Table:** 

Strains	Genotype	Original strain name	Original source	NBRP ID
DY7144	* h ^−^ can1-1 *	Kohli 10-389	Jürg Kohli	FY18665
DY49525	* h ^−^ can2-1 *	Kohli 10-391	Jürg Kohli	FY18666
LD331	* h ^+^*	–	Li-Lin Du	–
DY50489	* h ^+^*	–	Xiao-Hui Lyu	–
DY50490	* h ^+^*	–	Xiao-Hui Lyu	–
DY50491	* h ^+^*	–	Xiao-Hui Lyu	–
DY50492	* h ^+^*	–	Xiao-Hui Lyu	–
DY50493	* h ^+^ any1-R175C *	–	Xiao-Hui Lyu	–
